# *In Vitro* Anthelmintic Activity of Methanolic Extract from* Caesalpinia coriaria* J. Willd Fruits against* Haemonchus contortus* Eggs and Infective Larvae

**DOI:** 10.1155/2018/7375693

**Published:** 2018-11-29

**Authors:** X. De Jesús-Martínez, A. Olmedo-Juárez, J. Olivares-Pérez, A. Zamilpa, P. Mendoza de Gives, M. E. López-Arellano, S. Rojas-Hernández, A. Villa-Mancera, L. M. Camacho-Díaz, M. Cipriano-Salazar

**Affiliations:** ^1^Programa de Posgrado en Ciencias Agropecuarias y Gestión Local, FMVZ, Universidad Autónoma de Guerrero, Mexico; ^2^Centro Nacional de Investigación Disciplinaria en Parasitología Veterinaria, INIFAP, Mexico; ^3^Centro de Investigación Biomédica del Sur, Instituto Mexicano del Seguro Social, Argentina No. 1. Col. Centro, CP. 62790 Xochitepec, Morelos, Mexico; ^4^Facultad de Medicina Veterinaria y Zootecnia, Benemérita Universidad Autónoma de Puebla, Mexico

## Abstract

The aim of this study was to evaluate the* in vitro* lethal effect of a methanolic extract (ME) from* Caesalpinia coriaria* fruits against* Haemonchus contortus* eggs and infective larvae. The anthelmintic activity was assessed using the egg hatching inhibition assay (EHI) and the mortality test. The ME was assessed using five concentrations as follows: 6.15, 3.12, 1.56, and 0.78 mg/mL to eggs and 150, 100, 75, and 50 mg/mL to larvae, respectively. Ivermectin (5 mg/mL) was used as positive control and 4% methanol and distilled water were used as negative controls. The data of ovicidal and larvicidal effect were analyzed with a completely randomized design through ANOVA analysis using the general linear model (GLM) and lethal concentrations (LC_50_ and LC_90_) were estimated through a Probit analysis using the SAS program. A clear ME increased concentration dependence effect was observed in the EHI and mortality tests. The highest activity of the methanolic extract was observed at the highest concentration (P < 0.05) to obtain a similar effect to the positive control (ivermectin), with LC_50_ = 78.38 and 0.00064 mg/mL and LC_90_ =235.63 and 0.024 mg/mL, respectively, for larvae and eggs. The results indicate that the* C. coriaria* fruit ME possesses* in vitro* ovicidal and larvicidal properties (gallotannins: methyl gallate) against* H. contortus* that needs to be investigated more* in vivo *for the control of gastroenteric nematodes in ruminants.

## 1. Introduction

 Ruminants under grazing conditions in tropical and subtropical zones are exposed to several economically important parasite species. Among these the gastrointestinal nematodes (GIN), which affect the livestock industry worldwide, represent one of the most important parasites group.* Haemonchus contortus* is one the mots pathogenic parasites that affect the health of small ruminants [1]. The excessive use of chemical anthelmintics is leading to increased occurrence of anthelmintic resistance [2, 3]. In this context, the use of plant extracts and their secondary metabolites might represent a future alternative to chemical anthelmintics. Several studies have reported that some secondary compounds from arboreal leguminous express anthelmintic effects against livestock parasites [4–6].* Caesalpinia coriaria* Jacq Willd is a tree named as “Cascalote” in Mexico, and it is very widespread in the Tierra Caliente region of Guerrero. This plant produces a number of secondary metabolites like tannins and some flavonoids [7, 8]. Isolated compounds from this leguminous have shown medicinal properties such as antioxidant and anticancer activities [8]. Thus, this leguminous might be interesting to investigate its activity against parasitic stages, with the intention of using them in animals, since reports indicate that the fruits of this tree are consumed by ruminants in their grazing routes, without manifestations of intoxication symptoms [9].

Therefore, the objective of this study is to evaluate the* in vitro* anthelmintic activity of methanolic extract from* Caesalpinia coriaria* J. Willd fruits against eggs and infective larvae (L3) of the gastrointestinal parasite* H. contortus*.

## 2. Materials and Methods

### 2.1. Plant Material

*Caesalpinia coriaria* dried fruits (5000 g) were collected in the Tierra Caliente region of Guerrero, Mexico, located at 18° 20′ 30′′ NL and 100° 39′18′′ WL, and were dried to constant weight in a forced air oven at 45°C for 72 h. The dry material was ground using an electrical miller to reduce the fruit obtaining a 1 mm particle size.

### 2.2. Preparation of Methanolic Extract

Once ground, the dry material (100 g) was suspended in 2000 mL of methanol as extraction solvent, for 24 h at room temperature. The solvent methanol was used in the order to obtain both less and high polar compounds. The liquid solution was filtered using different filters (gauze, cotton, and filter paper), and the residual solvent was removed by distillation under reduced pressure with the help of a rotary evaporator (Buchi R-114) at 50°C, to obtain a semisolid extract, which was dried by lyophilization processes. Finally, the solvent-free dry extract (36 g) was obtained and stored at -40°C until its later use in the* in vitro* bioassays.

### 2.3. Identification of Bioactive Compounds of the Extract

The methanol extract of* C. coriaria* dried fruits was analyzed by high performance liquid chromatography (HPLC), using a Waters 2695 (Waters Corporation, USA) and a LC-F Supelcosil column (4.6 mm x 50 mm i.d., 5-*μ*m particle size; Sigma, Aldrich, Bellenfnote, USA) for chemical separation. The mobile phase consisted of an aqueous solution with 0.5% trifluoroacetic acid (Solvent A) and acetonitrile (Solvent B). The gradient system was as follows: 0-1 min, 0% B; 2-3 min, 5% B; 4-20 min, 30% B; 21-23 min, 50% B, 24-25 min, 80% B; 26-27 min 100% B; 28-30 min, 0% B. The retention rate was maintained at 0.9 mL/min, with an injection volume of 10 *μ*L. The absorbance was measured at 330 nm. The standards of gallic acid and methyl gallate (Sigma-Aldich®, USA) were used as reference standards.

### 2.4. Biological Material

#### 2.4.1. Harvest of Haemonchus Contortus Eggs

*Haemonchus contortus* eggs obtained from a donor ovine experimentally infected with infective larvae (L_3_) of the parasite were used (strain INIFAP, 350 L_3_/kg of BW of the animal). The eggs were concentrated by passing different sieves (200, 100, 75, and 37 *μ*m of diameter) and by density gradients with 40% sucrose. Egg recovery was performed according to the technique described by Coles et al. [10]. With the recovered eggs, an aqueous suspension was prepared at a concentration of 100 eggs per mL for use in the parasite egg hatching inhibition assay.

#### 2.4.2. Egg Hatching Inhibition Test (EHI)

The assay was performed using 96-well microtitration plates (n = 6). The treatments were the methanol extract at different concentrations (6.15, 3.12, 1.56, and 0.78 mg/mL), 4% methanol (Merck®, Germany) and distilled water as a negative control and ivermectin (5 mg/mL, Sigma-Aldrich®, USA) as a positive control. Fifty microliters of an aqueous suspension containing 100 ± 10 eggs of* H. contortus* were distributed in each well. Afterwards, 50 *μ*L aliquots of the extract and controls were added, with a final volume of 100 *μ*L per well. The plates were incubated for 48 hours at a temperature of 28°C. The egg hatching process was stopped by adding 10 *μ*L of Lugol solution. Finally, the total eggs or larvae of each well were counted and the percentage of inhibition of egg hatching (%EHI) was determined by the following formula: % EHI = [(number of eggs)/(number of larvae (L1+L2) + number of eggs)] *∗* 100, [10].

#### 2.4.3. Harvest of* Haemonchus contortus* Infective Larvae

Larvae (L_3_) were obtained from the donor sheep by daily collection (24 h). Fecal cultures were prepared by mixing the feces with polyethylene particles in plastic basins. Water was added to the fecal material and homogenized to obtain adequate oxygenation to promote better hatching of the eggs. The fecal cultures were covered with aluminum foil and incubated for 7 days at room temperature (25-31°C). The infective larvae were extracted of the fecal material using the Baermann funnel technique [11]. The L3 were cleaned by density gradient and centrifugation; the larvae were drawn with 0.187% sodium hypochlorite and washed with distilled water. Finally, they were used in the* in vitro* bioassays.

#### 2.4.4. *In Vitro* Larval Mortality Test (L_3_)

Microtiter plates (96-well) were used, where 50 *μ*L of the extract solutions were deposited (n = 6). The treatments were four concentrations of the extract (150, 100, 75, and 50 mg/mL), and two controls, one positive (C+) with 5 mg/mL of ivermectin and another negative (C-) with 4% methanol and distilled water. After each well, 100 L_3_ larvae were deposited in 50 *μ*L solution to complete a total volume of 100 *μ*L. Finally, the plates were incubated at 28°C, for 72 hours. Ten aliquots of 5 *μ*L were taken to count alive or dead larvae and with this the mortality percentage of larvae L_3_ (% ML_3_) was calculated by the following formula: % ML_3_ = [(number L_3_ dead/(number of L_3_ dead + number of live L_3_)] *∗* 100. The criteria of L3 mortality were assessed according to Olmedo-Juárez et al. [7] considering if mobility was not observed during 15-20 s. When larvae remained motionless but their aspect caused confusion about if they were dead or alive, a physical stimulus was applied touching their coat with a metal needle and the final decision was based on their motility.

### 2.5. Statistical Analysis

The results obtained were analyzed in a completely randomized design through ANOVA analysis using the general lineal model (GLM), with the following statistical model: *Y*_*ij*_ = *μ* + *T*_*i*_ + *ξ*_*ij*_, where *Y*_*ij*_ = inhibition of egg hatching and larval mortality; *μ* = general mean; *T*_*i*_ = effect of the concentration of the extract and controls, and *ξ*_*ij*_ = the random error of the treatments. The difference between means was compared with the Tukey test (P <0.05). Likewise, minimum (LC_50_) and maximum (LC_90_) lethal concentrations were determined using the PROBIT procedure of the statistical package [12].

## 3. Results and Discussion

### 3.1. Egg Hatch Inhibition Test

**Figure 1:**
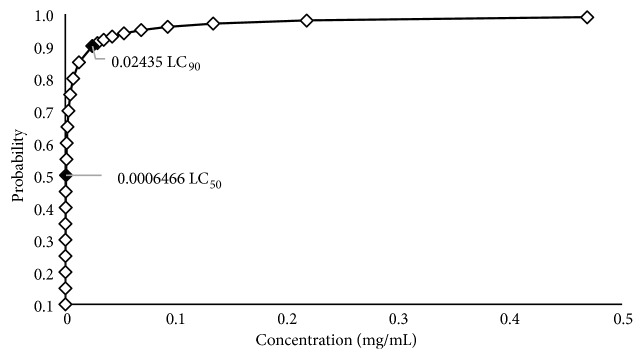
Lethal concentrations (LC) of the methanolic extract from* C. coriaria* J. Willd fruits to inhibit* H. contortus* eggs (24 h of* in vitro* exposure).

Table 1 and Figure 1 show the lethal concentrations 50 and 90 produced by the* C. coriaria* fruit methanolic extract on* H. contortus* eggs after 48 h of exposure. The LC_50_ and LC_90_ values of the ovicidal effect were 0.0006 and 0.0243 mg/mL, respectively. On the other hand, the results of the EHI percentages, the methanolic extract showed an ovicidal activity close to 99% with the concentrations of 1.56 and 0.78 mg/mL, (P <0.05), similar to the positive control. Boubaker et al. [13] report anthelmintic activity of two extracts, aqueous and ethanolic, respectively, evaluated against* H. contortus* eggs, where they obtained results at a lethal concentration (LC_50_) of 0.368 *μ*g/mL for the ethanolic extract and for the aqueous extract (LC_90_) of 6.344 *μ*g/mL. Likewise Pérez-Pérez et al. [2] reported* in vitro* activity of the methanolic extract of* Gliricidia sepium* leaves, and the percentages of effectiveness found were 27.7%, 46.2%, and 49.7% inhibition at 125, 250, and 500 *μ*g/mL, respectively, with an LC_50_ of 394.96 *μ*g/mL. Castillo-Mitre et al. [4] report poor ovicidal activity against* H. contortus* of the methanolic fraction in extracts made from* Acacia cochliacantha* leaves; however, they describe a strong action of the organic fraction at initial doses of 1.56 *μ*g/mL and LC_50_: 0.33 *μ*g/mL and LC_90_: 0.85 *μ*g/mL. The comparative analysis of these results clearly highlights that the methanol extract obtained from the fruits of* C. coriaria* was more active to inhibit eggs hatching of the parasite, because 99% inhibitions were obtained with the lowest doses and the activity was accentuated up to 100% of the inhibition with the higher concentrations. This reflects the potential use of the extract for the control of parasitic infestations in the livestock industry.

### 3.2. Mortality Test of Infective Larvae (L3)

**Figure 2:**
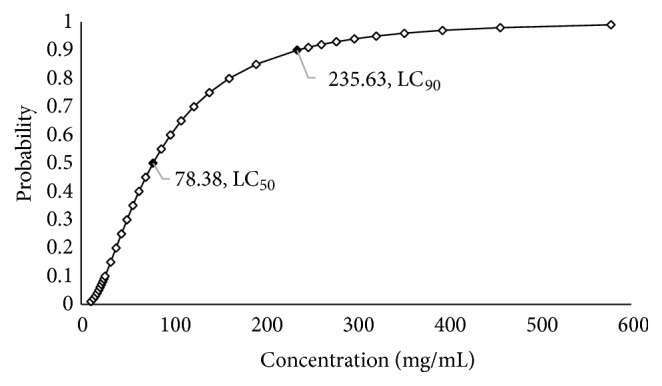
Lethal concentrations (LC) of the methanolic extract of* C. coriaria* J. Willd fruits for infecting larvae (L_3_) of* H. contortus* (72 h of* in vitro* exposure).

Table 2 show the results of the mortality percentages of* H. contortus* L_3_ exposed to the extract at the different concentrations and their controls, respectively. An effect of 78.3 and 72.3% against L_3_ of* H. contortus* was observed at the highest concentrations of 100 and 150 mg/mL, respectively. The extract of* C. coriaria* had a larvicidal effect (L_3_) superior to that observed in the negative control, but lower than the positive control (P <0.05). The lethal concentrations observed were LC_50_ = 78.38 mg/mL and LC_90_ = 265.63 mg/mL; schematically in Figure 2 it can be seen that to increase the larvicidal effect from 50 to 90% or greater, it is required about three times the concentration of the extract.

The results of the effect of the fruit extract of* C. coriaria* on* H. contortus* larvae are similar to those reported by other researchers. Olmedo-Juárez et al. [5] reported a larvicidal effect (*H. contortus*) greater than 70% at a dose of 150 mg/mL with an extract made from* Acacia cochliacantha* leaves and an LC_50_ and LC_90_ of 127.3 and 177.8 mg/mL, respectively. Also Chan-Pérez et al. [14, 15] observed that* H. contortus* larvae after being exposed to an acetonic: aqueous extract of *A. pennatula* and* O. viciifolia* at concentrations of 600 *μ*g/mL showed evident lesions such as separation of the cuticle and the internal structures including the pharynx, bulb, and intestinal cells and when they were exposed to doses of 5000 *μ*g/mL the cuticle of the larvae was clearly swollen and the internal structures (pharynx, bulb, and intestine) were not distinguishable. The results also show that the larvicidal activity of the extract depends directly on the concentration dose. In the case of the methanolic extract of* C. coriaria* fruits it is necessary to consider high concentrations >300 mg/mL to obtain a larvicidal effect superior to the 90%; this same tendency has been described by Alonso-Díaz et al. [16] and Alemán et al. [17] in studies developed with extracts from other plants. However, there are other factors to consider in the use of plant extracts as a resource to control parasitic diseases, such as the type of parasitic isolation where Calderon-Quintal et al. [18], Alonso-Díaz et al. [19], and Vargas-Magaña et al. [20] observed differences in the susceptibility of* H. contortus* larvae from mainly Mexican and French isolates, which can be attributed to certain tolerance developed by the parasite.

### 3.3. Identification of Bioactive Compounds by HPLC

**Figure 3:**
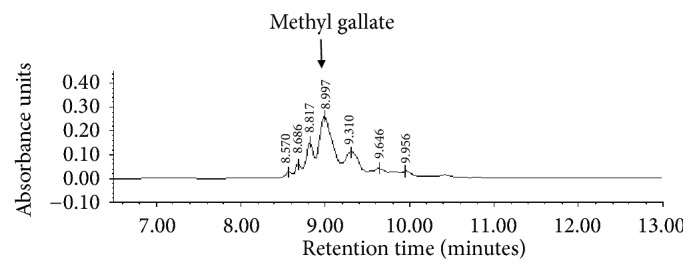
Secondary compounds identified in the methanolic extract of* C. coriaria* J. Willd fruits (HPLC analysis).

In the methanolic extract of* C. coriaria* dried fruits, methyl gallate was identified as a major compound and gallate derivatives as other compounds (Figure 3). These compounds could be responsible for ovicidal and larvicidal activity against* H. contortus*. However, future studies with this plant, through chemicals bioguided assay to identify the responsible metabolite of the anthelmintic activity, are necessary.* Caesalpinia coriaria *is a tree with multiple uses in traditional medicine in Mexico [8]. Phytochemical reports detected that the fruits of* C. coriaria* have a high content of phenolic compounds [2, 8] which have diverse properties such as anti-inflammatory, cicatrizing and apoptosis in cancer cells [8]. Additionally Mi-Sun et al. [21] reported activity of these phenolic compounds against bacteria. In this study it was demonstrated that the gallotannins identified in the methanolic extract of the* C. coriaria* fruits had anthelmintic properties for the* in vitro* control of the eggs and larvae stages of* H. contortus*. Developed publications agree that the condensed tannins present in plants have activity against gastroenteric parasites of ruminants [12, 22]. However, synergic effect of tannins has also been reported with other compounds such as flavonoids in the control of parasites [9, 17]. These results suggest that in any control program of parasites, where it is intended to use tree extracts by their nematicidal effect, it is necessary to consider the concentration dose, the type of bioactive compound, and the parasitic isolate, as main factors that determine the anthelmintic efficacy.

## 4. Conclusions

The* in vitro* ovicidal and larvicidal activity of the methanolic extract of* Caesalpinia coriaria* J. Willd fruits against* H. contortus* was demonstrated, and phenolic compounds such as methyl gallate and its derivatives were identified as possibly responsible for the anthelmintic effect. In addition, the ovicidal effect was observed at the lower concentrations of the extract, while the larvicidal effect was observed at the highest concentrations, which indicated that the effect against the stages of the parasite depends on the concentration dose. These results justify continuing the investigation on* Caesalpinia coriaria* J. Willd fruits* in vivo* under controlled conditions to verify if the activities recorded* in vitro* could be reproduced* in vivo* in sheep infested with* H. contortus.*

## Figures and Tables

**Table 1:** Egg hatching inhibition (% EHI) of *Haemonchus contortus* exposed to a methanolic extract made with *Caesalpinia coriaria* fruits.

Treatments	Means of eggs and larvae (L1 or L2) recovered		% EHI
	Eggs	L1+L2	
Methanol 4 %	06	34	15.00^b^
Ivermectin 5 mg/mL	91	0	100^a^
Water (H_2_O)	03	64	4.47^c^
*C. coriaria* fruit extract (mg/mL)			
6.15	121	0	100^a^
3.12	90	0	100^a^
1.56	90	1	98.90^a^
0.78	117	0	100^a^
Variation coefficient			3.35
Standard error of means			0.13

^abc^ Means with different letter in the same column statistically differ, Tukey (P <0.05).

**Table 2:** Mortality percentages of infective larvae (L_3_) of *Haemonchus contortus* exposed to a *Caesalpinia coriaria* methanolic extract at different concentrations.

Treatments	Means infective larvae (live or dead) recovered		% Mortality
	Live	Dead	
Methanol 4 %	3	63	4.54^d^
Ivermectin 5 mg/mL	0	80	100^a^
*C. coriaria *fruits methanolic extract (mg/mL)			
150	14	38	73.07^b^
100	14	51	78.46^b^
75	49	29	37.19^c^
50	52	30	36.58^c^
Variation coefficient			10.4
Standard error of means			0.35

^abc^ Means with different letter in the same column statistically differ, Tukey (P <0.05), LC = lethal concentration.

## Data Availability

The data used to support the findings of this study are available from the corresponding author upon request.
